# Bearing the burden of austerity: how do changing mortality rates in the UK compare between men and women?

**DOI:** 10.1136/jech-2022-219645

**Published:** 2022-10-04

**Authors:** David Walsh, Ruth Dundas, Gerry McCartney, Marcia Gibson, Rosie Seaman

**Affiliations:** 1 Glasgow Centre for Population Health, Glasgow, UK; 2 MRC/CSO Social and Public Health Science, University of Glasgow, Glasgow, UK; 3 College of Social Sciences, University of Glasgow, Glasgow, UK; 4 Clinical and Protecting Health, Public Health Scotland Glasgow Office, Glasgow, UK

**Keywords:** MORTALITY, POLICY, Health inequalities, DEMOGRAPHY

## Abstract

**Background:**

Mortality rates across the UK stopped improving in the early 2010s, largely attributable to UK Government’s ‘austerity’ policies. Such policies are thought to disproportionately affect women in terms of greater financial impact and loss of services. The aim here was to investigate whether the mortality impact of austerity—in terms of when rates changed and the scale of excess deaths—has also been worse for women.

**Methods:**

All-cause mortality data by sex, age, Great Britain (GB) nation and deprivation quintile were obtained from national agencies. Trends in age-standardised mortality rates were calculated, and segmented regression analyses used to identify break points between 1981 and 2019. Excess deaths were calculated for 2012–2019 based on comparison of observed deaths with numbers predicted by the linear trend for 1981–2011.

**Results:**

Changes in trends were observed for both men and women, especially for those living in the 20% most deprived areas. In those areas, mortality increased between 2010/2012 and 2017/2019 among women but not men. Break points in trends occurred at similar time points. Approximately 335 000 more deaths occurred between 2012 and 2019 than was expected based on previous trends, with the excess greater among men.

**Conclusions:**

It remains unclear whether there are sex differences in UK austerity-related health effects. Nonetheless, this study provides further evidence of adverse trends in the UK and the associated scale of excess deaths. There is a clear need for such policies to be reversed, and for policies to be implemented to protect the most vulnerable in society.

**WHAT IS ALREADY KNOWN ON THIS TOPIC**

- Concerning changes to mortality rates—a stalling of improvement overall, with increasing death rates among the poorest—have been observed across the UK since the early 2010s.
- A growing evidence base has attributed these changes to UK Government’s austerity policies which have cut both the income of the poorest and a range of important public services.
- There is evidence that the financial impact of such austerity policies has been greater for women than men.

**WHAT THIS STUDY ADDS**

- We quantify the scale of excess deaths observed in Scotland, England and Wales since 2012.
- In comparison with what was predicted (based on previous trends), a conservative estimate of approximately 335 000 additional deaths occurred between 2012 and 2019.
- There is some evidence that among more deprived populations, female mortality rates have worsened to a greater degree.

**HOW THIS STUDY MIGHT AFFECT RESEARCH, PRACTICE OR POLICY**

- The UK Government needs to understand the immensely damaging health impact of its austerity policies to date.
- There is an urgent need for these policies to be reversed, and for the UK Government to instead implement measures to protect the most vulnerable in society.

## Introduction

There is clear evidence of adverse changes to mortality rates in the UK from the early 2010s onwards: a slow-down in the rate of improvement overall, alongside increasing death rates among more socioeconomically deprived populations; inequalities have widened considerably as a consequence of the latter.^1–9^ These changes predate the COVID-19 pandemic and are important context for understanding the scale of pandemic-related inequalities.^10 11^

Although a number of different contributory factors were initially proposed, a considerable body of evidence now demonstrates that UK Government’s ‘austerity’ policies are the main cause of these pre-pandemic changes.^12–17^ This includes a recently published, large-scale, critical assessment of all the relevant evidence.^18^ Such policies—introduced from 2010 onwards, and following ‘the great recession’ of 2008—have removed billions of pounds from both social security and vital services, and have thus particularly impacted on poorer, more vulnerable, populations.^10 15 18^ Similar adverse effects of austerity measures on population health have been seen in other high-income countries.^19–22^

There is also evidence that the reductions in social security income and loss of services have disproportionately affected women in the UK. This is for a number of important reasons including: more women being in receipt of social security payments in the first place; the disproportionate effects of cuts on particular, female-dominated, groups such as lone parents; the contraction in public sector jobs where women are likely to be employed; and inequalities in caring responsibilities (and the associated need for local government services and social care in particular).^23–29^ Among the elderly, the fact that more women live alone and are unable to share financial burdens may also be relevant.^30^ However, it is unclear whether the *mortality* impact of austerity has also been worse for women. Some recent descriptive trends supported this hypothesis, with adverse changes in all-cause mortality seemingly occurring earlier for women (around 2010–2011) than men (around 2012) in some UK countries and cities.^4^ In contrast, previous analyses of Scottish trends suggested similar turning points for both sexes.^3^ Given that uncertainty, the overall aim of this study was to examine whether there are differences in trends between men and women in Great Britain which might support the hypothesis of a greater health impact of austerity on women. Specifically, we sought to statistically test whether there are differences when all-cause mortality trends changed, and to quantify the number of sex-specific deaths that have been observed in the past decade compared with what was expected given previous trends.

## Methods

### Data sources

All-cause mortality and matching population denominator data were obtained for Scotland (from the National Records of Scotland) and England (from the Office of National Statistics (ONS)) by 5-year age band, sex and year for the period 1981–2019. Data were additionally stratified by area deprivation quintile for the years 2001–2019, based on the Scottish Index of Multiple Deprivation^31^ and the English equivalent (Index of Multiple Deprivation).^32^ As previously described,^4^ although these indices differ in terms of some of the individual variables included, and the spatial scale at which they are constructed, these differences are outweighed by the similarities of their composition (including the use of the same principal ‘data domains’) and the methodologies employed in their calculation. Thus, while the absolute values of the two indices cannot be directly compared, their similarity enables comparable overviews of inequality within each country. Both indices have been updated over time; thus, different versions were used to cover different time periods. Full details are included in the online supplemental table 1. Unfortunately, separate data for Wales, stratified by the equivalent Welsh deprivation measure, were not available at the time of undertaking the analyses.

10.1136/jech-2022-219645.supp1 Supplementary data

For the analysis of excess deaths, data for England and Wales (rather than England alone) were obtained from ONS.

### Statistical analyses

European age-standardised mortality rates (ASMRs) per 100 000 population were calculated using the 2013 European Standard Population.^33^ As background to the main analyses, time trends were examined using 3-year rolling averages, with changes in rates calculated between 2010/2012 (when austerity policies were first introduced) and 2017/2019. Using single-year time points, segmented regression analyses were then run to identify break points in trends between 1981 and 2019: this was limited to the identification of one break point; where the identified break point predated 2010, further analyses were run restricting identification of points to after 2010 only. All analyses were stratified by country (Scotland, England), sex, age (all ages, 0–64 years) and deprivation quintile (comparing rates for people living in the most and least deprived 20% of areas in each country).

To calculate the total excess deaths for the period 2012–2019, the predicted trend of ASMRs was calculated, based on the linear trend for 1981–2011. These predicted ASMRs were applied to the actual population for each year to obtain ‘expected’ deaths (ie, if the trend from 1981 to 2011 had continued for 2012–2019). ‘Observed’ deaths were similarly calculated by applying the age-standardised rates to the population for that year. However, these will differ from the real number of observed deaths in each year because they apply *standardised* rates, thereby accounting for any trends that can be attributed to changes in the underlying age structure of the population. The excess deaths were calculated as ‘observed’ deaths minus expected deaths. Year 2012 was chosen as the cut-off on the basis of previous analyses of trends.^3^

### Results

**Figure 1:**
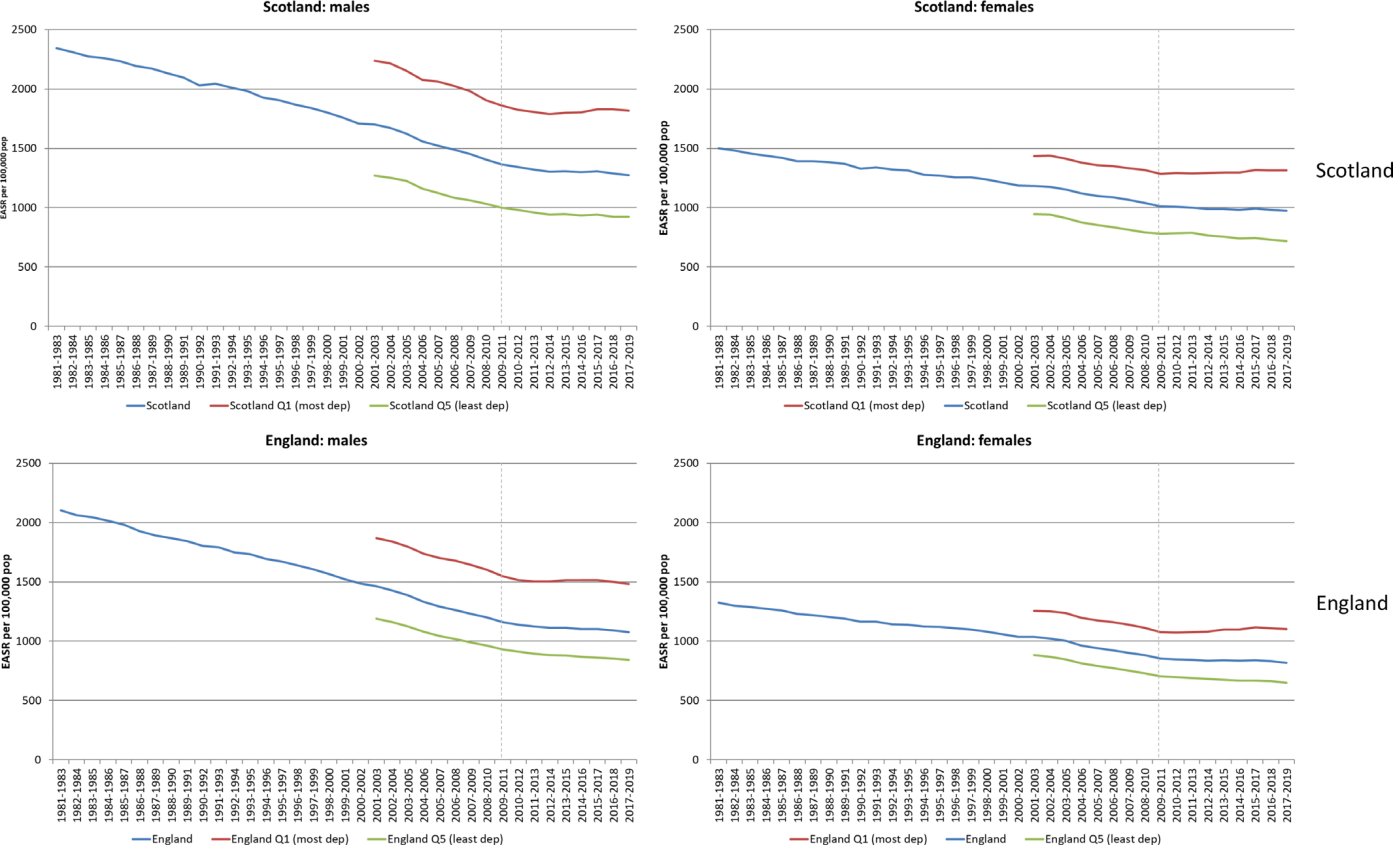
European age-standardised mortality rates (EASRs) per 100 000 population (3-year rolling averages) 1981–2019 for men and women: Scotland, England and their 20% most and least deprived populations.

Figure 1 shows trends in ASMRs for men and women in Scotland and England, comparing overall rates and those of the most and least deprived deprivation quintiles. The data are presented as 3-year rolling averages, with the dotted line signifying the year of implementation of UK Government’s austerity policies. The data presented are for all ages; similar trends for 0–64 years are shown in the online supplemental figure 1. For all ages (figure 1), there is some suggestion of an earlier change in rates for women compared with men; however, for premature mortality (online supplemental figure 1), the point of change looks similar in both countries (and is much clearer in Scotland than England). These points are discussed further below.

**Figure 2:**
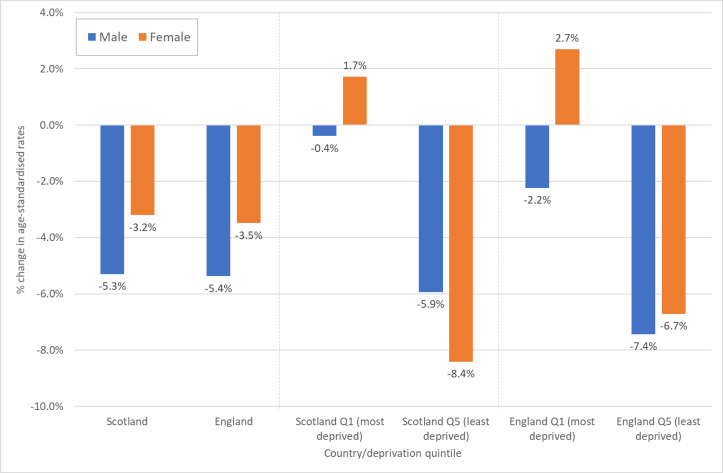
Percentage change in European age-standardised mortality rates (all ages), 2010/2012–2017/2019: Scotland, England and their 20% most and least deprived populations.

Figure 2 quantifies the changes in rates between 2010/2012 and 2017/2019 shown in figure 1. The most notable difference between men and women is among those living in the most deprived quintile of each country where mortality rates increased over the period for women, but reduced slightly for men. However, for premature mortality (online supplemental figure 2), rates of change were similar for men and women: for example, among the most deprived populations, rates increased among both in Scotland (+6.0% for men, +6.7% for women), and declined slightly among both in England (−2.8% and −2.7%, respectively).

**Figure 3:**
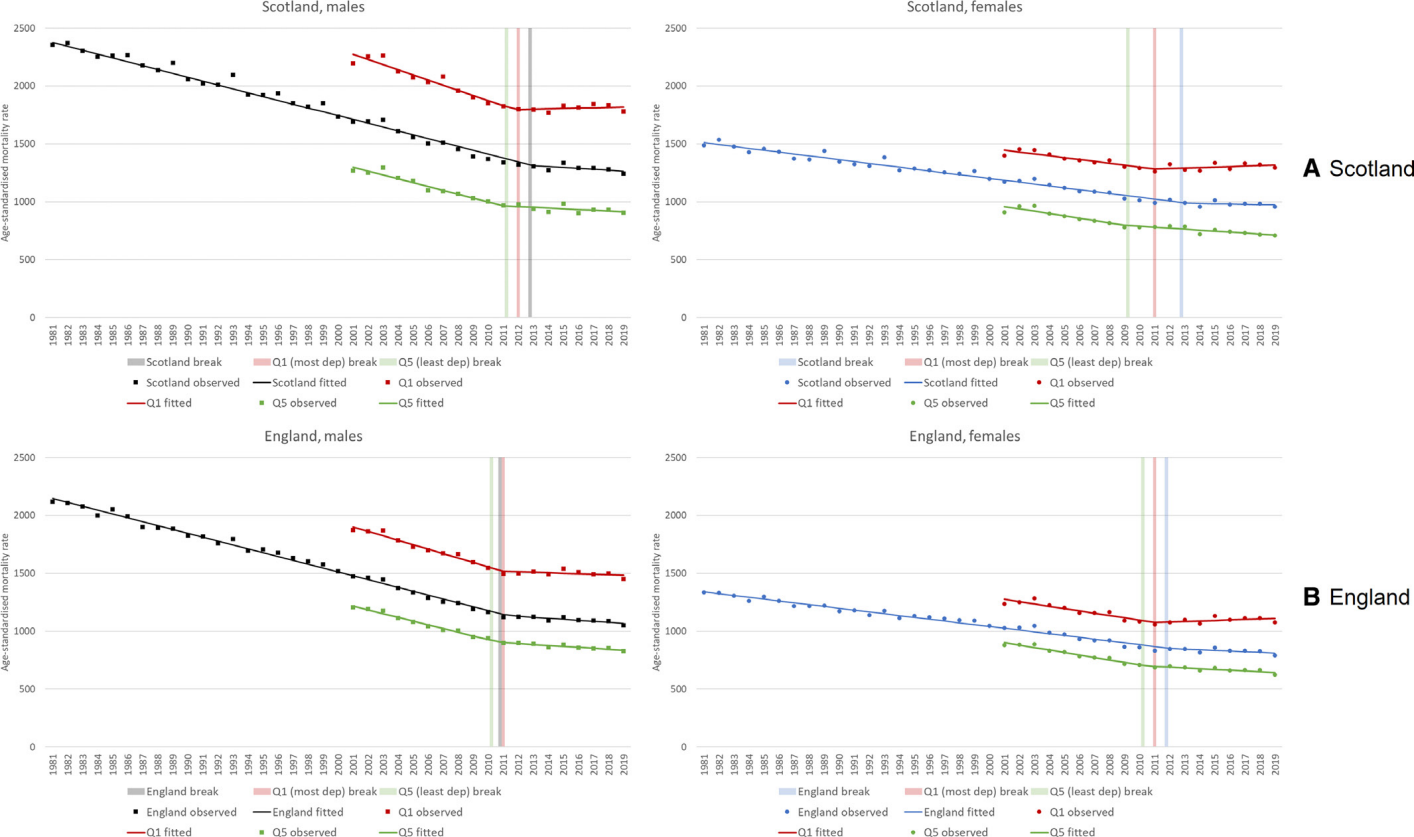
European age-standardised mortality rates (all ages), 1981–2019, (A) Scotland and (B) England and its 20% most and least deprived populations: observed rates, fitted regression line and break points.

Figure 3 shows these trends in a different manner, presenting not 3-year rolling averages, but annual rates alongside both fitted linear trends and the identified break points from the segmented regression analyses. Data are for all ages. Note that the precise values of all break points, including 95% CIs, are shown in table 1. Changes are apparent in all the trends, but most noticeably among the most deprived populations. The break point for Scotland as a whole is 2013 for both men and women; in England it is 2011 for men and 2012 for women. Among the most deprived 20% of both countries, there is again very little difference in break points between men and women: 2011 for both men and women in England; 2011 and 2012 for women and men, respectively, in Scotland.

**Table 1:** Summary of break points (with 95% CIs) identified in segmented regression analyses

Country/ deprivation quintile	All ages		0–64 years	
	Male	Female	Male	Female
Scotland	2013.0 (2010.3 to 2015.7)	2013.3 (2009.4 to 2017.1)	2012.7 (2011.1 to 2014.3)	2003.7 (2001.1 to 2006.3)†
England	2011.3 (2009.7 to 2012.9)	2012.1 (2008.1 to 2016.0)	1999.6 (1997.8 to 2001.4)*	2001.5 (1999.5 to 2003.5)‡
Scotland quintile 1 (most deprived)	2011.8 (2010.1 to 2013.4)	2011.0 (2008.4 to 2013.6)	2012.9 (2012.0 to 2013.8)	2013.4 (2011.9 to 2014.9)
Scotland quintile 5 (least deprived)	2011.0 (2009.0 to 2013.1)	2009.0 (2004.9 to 2013.1)	2011.0 (2007.3 to 2014.7)	n/a§
England quintile 1 (most deprived)	2011.0 (2009.6 to 2012.4)	2011.0 (2008.9 to 2013.0)	2012.0 (2010.5 to 2013.5)	2012.0 (2010.1 to 2013.9)
England quintile 5 (least deprived)	2010.7 (2009.7 to 2011.7)	2010.5 (2008.5 to 2012.4)	2013.7 (2012.5 to 2014.8)	2013.4 (2012.0 to 2014.8)

*When analysis limited to break point after 2010, break point=2012.0.

†When analysis limited to break point after 2010, break point=2013.9.

‡When analysis limited to break point after 2010, break point=2012.2.

§No break point identified.

For analyses of premature mortality (online supplemental figure 3), pre-austerity break points were identified at the country level: 2000 (men in England), 2001 (women in England) and 2004 (women in Scotland). When analyses were restricted to identify any post-2010 changes in trends, break points were identified in 2012 (England) and 2013 (Scotland); however, there were few differences between men and women. Break points for the same years were noted for the most deprived populations, with again few differences between men and women.

Table 2 presents the results of the analysis of excess deaths for Scotland, England and Wales. These are for deaths at all ages; the equivalent figures for deaths <65 years are shown in online supplemental table 2. This suggests that across Great Britain, almost 335 000 more deaths occurred in the period 2012–2019 than would have been predicted on the basis of previous trends between 1981 and 2011. Over one-third of these were deaths under the age of 65 years. However, contrary to the hypothesis under investigation, the majority of all these deaths (66% in Scotland, 75% in England and Wales) were for men. Expressed as the percentage of the total ‘observed’ deaths, the excess was 9.0% and 3.7% for men and women, respectively, in England and Wales, with the equivalent figures for Scotland being 4.0% and 2.6%. However, for premature deaths only, the excess in Scotland was slightly higher for women: 11.8% compared with 9.6%; in England and Wales, the figure for women (13.3%) was lower than for men (17.7%). Note that these analyses predate the COVID-19 pandemic. Charts showing the observed and predicted mortality rates by sex and country are presented in online supplemental figure 4.

**Table 2:** ‘Observed’, expected and excess deaths by county and sex, 2012–2019 (all ages)

		Male				Female			
		‘Observed’	Expected	Excess (n)	Excess (%)	‘Observed’	Expected	Excess (n)	Excess (%)
Scotland	2012	34 741	35 560	−818	−2.4	28 168	28 019	149	0.5
	2013	34 569	34 809	−240	−0.7	27 434	27 609	−175	−0.6
	2014	33 751	34 058	−307	−0.9	26 589	27 250	−661	−2.5
	2015	35 600	33 352	2248	6.3	28 229	26 902	1327	4.7
	2016	34 642	32 676	1966	5.7	27 344	26 580	764	2.8
	2017	34 915	31 936	2978	8.5	27 683	26 185	1499	5.4
	2018	34 697	31 137	3559	10.3	27 707	25 764	1943	7.0
	2019	33 746	30 398	3347	9.9	27 115	25 397	1718	6.3
	Total 2012–2019	276 661	263 926	12 735	4.6	220 269	213 705	6564	3.0
England and Wales	2012	322 159	318 569	3590	1.1	245 470	247 532	−2062	−0.8
	2013	324 133	311 410	12 723	3.9	245 912	244 282	1630	0.7
	2014	316 467	304 536	11 932	3.8	238 820	241 309	−2489	−1.0
	2015	329 380	297 652	31 729	9.6	252 860	238 276	14 583	5.8
	2016	324 553	290 791	33 762	10.4	247 144	235 166	11 977	4.8
	2017	325 405	282 826	42 580	13.1	248 207	231 685	16 523	6.7
	2018	326 538	274 806	51 731	15.8	250 005	228 146	21 859	8.7
	2019	316 223	266 413	49 809	15.8	239 547	224 396	15 151	6.3
	Total 2012–2019	2 584 857	2 347 002	237 855	9.2	1 967 964	1 890 791	77 173	3.9

Expected deaths based on linear trend 1981–2011; excess deaths shown as percentage of observed deaths.

## Discussion

### Overall findings and implications

These analyses add to the existing evidence of worrying changes to mortality trends in the UK since the early 2010s. Compared with what previous trends predicted, an additional c.335 000 deaths were observed across Scotland, England and Wales between 2012 and 2019. However, contrary to the hypothesis under investigation, the majority of these deaths were for men. Furthermore, analyses of trends showed very few differences in break points between men and women. However, among those living in the most deprived 20% of areas in Scotland and England, mortality rates between 2010 and 2019 increased to a greater degree among women compared with men.

Comparisons of projected and observed rates are subject to a number of uncertainties, and thus we must be cautious in our interpretation of the precise figures shown here. Nonetheless, both the scale of excess mortality and direction of trends are hugely concerning (and other analyses suggest that our use of 1981 as the base year for the linear trend is likely to have produced a conservative estimate of the number of excess deaths).^34^ Given that these changes have been largely attributed to UK Government’s austerity policies, it is of paramount importance that the impact of such policies is understood, and that future policy seeks to support rather than damage population health in the UK.

### Strengths and weaknesses

Strengths of this study include the use of data for the whole populations of Scotland and England (and, for one set of analyses, also Wales) rather than samples. We analysed long-term trends covering almost 40 years. Mortality is a robust indicator of population health that is not subject to some of the uncertainties and potential biases associated with self-reported measures.^35 36^ That said, the use of such mortality data is clearly also a limitation: the impact of increased poverty and loss of services may be better understood using different measures on the well-understood causal pathways between social determinants and health outcomes. In addition, we only analysed all-cause mortality rather than specific causes. As stated, projections of mortality rates using simple linear trends can be problematic, and the results must be interpreted cautiously.^37^ The use of area-based deprivation measures can be problematic as they are based on ecological averages which may not apply to all individuals within them (ie, not all deprived populations live in the most deprived quintile).^38 39^ Finally, although the Scottish and English deprivation indices are very similar, they use different individual variables and are constructed at different spatial scales, and thus precise values are not directly comparable.

### Relevance to other studies

Although our analyses have produced mixed results in terms of the main hypothesis under investigation, the worse mortality experience of women living in the most deprived neighbourhoods might well reflect a more adverse, and gendered, financial impact of government policy. UK Government’s austerity cuts to social security payments and eligibility, as well as to important services, have been shown in a number of analyses to affect women more than men. This is for a number of reasons. First, women make up the majority of social security recipients in the UK, and have thus been more affected by the enormous cuts in spending in that area;^23–26 40 41^ that said, some of this has been contested as it can be difficult to disentangle impacts on an individual’s income from that of the broader household.^27 28^ Second, some individual social security cuts have disproportionately affected particular groups: lone parents (the vast majority of whom are female); single pensioners (more of whom are women) and people with disabilities (many of whom are looked after by female carers).^23–27 40 41^ Third, women are more likely to be poorer and on lower incomes—this is linked to inequalities in the labour market and in the division of caring responsibilities—and the cuts have been shown to be highly regressive, impacting most on those low-income groups.^23–26^ Fourth, as caring responsibilities (eg, for children, elderly and disabled people) are undertaken much more by women, they are not only affected by reductions in income, but also by cuts to, and loss of, public services that support such caring activities.^25 26 40 41^ Fifth, as the majority of public sector employees (especially part-time employees) in the UK are female, women are more likely to have been affected by both the public sector pay freezes that were introduced, and by public sector job losses.^25 26 40^ A range of other factors have been cited including the roll-out of the ‘Universal Credit’ social security benefit (as it disincentivises second income earners in a household, the vast majority of whom are female),^40^ cuts to legal aid/advice which have discouraged the challenging of discriminatory employment practices affecting women (eg, pregnancy/maternity discrimination),^41^ and cuts to specific services such as those for young children and in relation to gender-based violence.^25 26^

There have also been some attempts to quantify the differential financial impacts of austerity on men and women. For example, detailed analyses by the Equality and Human Rights Commission showed that as a result of changes to direct taxes and social security payments, women lost *on average* approximately £400 per year between 2010 and 2018 compared with c.£30 per year loss for men. However, that average figure varied enormously across both the income spectrum and different population groups: for example, women in the second and third lowest income deciles lost c.£1500 and c.£1100 per annum, respectively, compared with c.£1100 and c.£600 losses for men; similarly, among those aged 35–44 years, the average annual loss to women (c.£2200) was around four times higher than the equivalent loss to men (c.£550). The latter difference is linked to the average age of lone parents: and lone parents in the bottom income quintile were estimated to have lost around 25% of their entire annual income.^23^ Other, related, analyses have highlighted the intersectionality of gender, poverty and also ethnicity in assessing and quantifying the impact of the cuts. For example, among the poorest third of the population, white women lost 11% of their income (compared with 8% of equivalently poor men); however, the equivalent figures for black ethnic groups were 14% and 9%, respectively, and for Asian groups it was 19% and 10%.^25^

In terms of the gendered health impact of austerity, ecological analyses of mortality trends have produced mixed findings, although particularly adverse trends for socioeconomically deprived women have been demonstrated in several. Decomposition analyses of Scottish data suggested that changes in rates between 2000/2002 and 2015/2017 were slightly more pronounced among men than women;^7^ however, in comparable analyses for England over the period 2001–2016 (which had a particular focus on inequalities), particularly concerning trends for women were highlighted: widening inequalities overall, with a stalling of female life expectancy in the third to fifth most deprived deciles of the population, and declining life expectancy in the most deprived two deciles.^9^ A similar greater widening of life expectancy inequalities for women compared with men was shown for Wales between 2002 and 2018.^5^ These findings tally with our own deprivation-stratified analyses, and also with those of Rashid *et al* whose analyses for England showed falls in female life expectancy in almost one-fifth (18.7%) of the country’s small spatial units; the equivalent figure for men was 11.5%.^6^

Of studies which examined the association between austerity measures and mortality outcomes, some did not stratify by sex,^14 15 42^ while others showed broadly similar results for men and women.^12 13 43 44^ For example, Alexiou *et al* demonstrated a negative impact of local authority funding cuts on life expectancy in England, and for life expectancy *at birth*, the results were similar for both sexes (although the impact on life expectancy at age 65 years was slighter greater for women).^13^ Similarly, modelling analyses by Richardson *et al* for Scotland showed that reductions in life expectancy were associated with tax and social security changes, but the results were similar for men and women.^12^

Analyses of austerity-related trends in other health outcomes also present a mixed picture in terms of differences between sexes. Healthy life expectancy (which combines mortality data with information on self-assessed general health) decreased by 2 years in Scotland following implementation of austerity, but the decline was similar for men and women.^45^ However, Thomson *et al* showed that following the introduction of austerity, levels of poor mental health among those aged 25–64 years old in England worsened to a greater degree among women than men^46^; and although trends were only analysed up to 2014, subsequent analyses of the same data over a longer period (to 2018) showed worsening mental health among both men and women, but still to a greater degree among women.^47^ In contrast, Wickham *et al*, investigating the effect of one component of austerity on mental health—the introduction of the ‘Universal Credit’ social security benefit—showed similar adverse effects for men and women.^48^

Darlington-Pollock *et al* sought to quantify the excess numbers of deaths observed in England between 2010 and 2018.^49^ Their estimate of the number of excess deaths for England only (c.232 000) is of a similar magnitude to our estimate for England and Wales between 2012 and 2018 (c.250 000) (table 2). However, in contrast to our findings, the authors suggested that the excess deaths were much more evenly split between men and women. While there are differences between the studies in the methodological approaches taken (eg, Darlington-Pollock *et al*’s use of ONS 2010-based population projections as the basis for calculating the expected mortality rate by age, sex and area, geographical coverage, the use of national vs subnational data and the application of age standardisation in our study), further investigation would be required to establish the reasons for the disparity between the two studies.

The more adverse recent trends in premature (0–64 years) mortality observed in Scotland compared with England (for both men and women) shown in online supplemental figures 1 and 2 are largely explained by greater increases in drug-related deaths in Scotland in this period^18 50^; however, those trends have themselves been exacerbated by the same UK Government’s austerity policies discussed here.^15 18^ Finally, the reasons for the earlier (pre-austerity) breaks in the premature mortality trends (also shown in online supplemental figure 2) are unclear and are worthy of further investigation.

## Conclusions

It remains unclear if the well-evidenced health effects of the UK Government’s austerity programme have been more detrimental for female than male mortality, as had been hypothesised. While there is some supporting evidence for outcomes such as poor mental health, and while some of the analyses presented here suggest this may be the case for mortality rates among those living in more deprived areas, further work in this area is clearly required (and it will also be important to monitor these mortality trends post-pandemic). Nonetheless, this study adds to the growing evidence of deeply worrying changes to mortality trends in the UK—particularly among more socioeconomically deprived populations—which have been largely attributed to government policy. There is a clear and urgent need, therefore, for such harmful policies to be reversed, and instead for the UK Government to implement measures to protect the most vulnerable in society.

## Data Availability

No data are available.
