# Supplementary material for: Bearing the burden of austerity: how do changing mortality rates in the UK compare between men and women?
Source: J Epidemiol Community Health. 2022 Oct 4;76(12):1027–33. doi: 10.1136/jech-2022-219645 (PMC9664129; doi:10.1136/jech-2022-219645)

Table S1. Versions of the Scottish and English Indices of Multiple Deprivation (SIMD and (E)IMD) used in the analyses.

Scottish Index of Multiple Deprivation (SIMD)			(English) Index of Multiple Deprivation (IMD)	
Version	Analysis years		Version	Analysis years
SIMD 2004	2001-04		IMD 2004	2001-05
SIMD 2006	2005-07		IMD 2007	2006-08
SIMD 2009	2008-10		IMD 2010	2009-13
SIMD 2012	2011-13		IMD 2015	2014-17
SIMD 2016	2014-17		IMD 2019	2018-19
SIMD 2020	2018-19			

Walsh D, *et al. J Epidemiol Community Health* 2022; 76:1027–1033. doi: 10.1136/jech-2022-219645

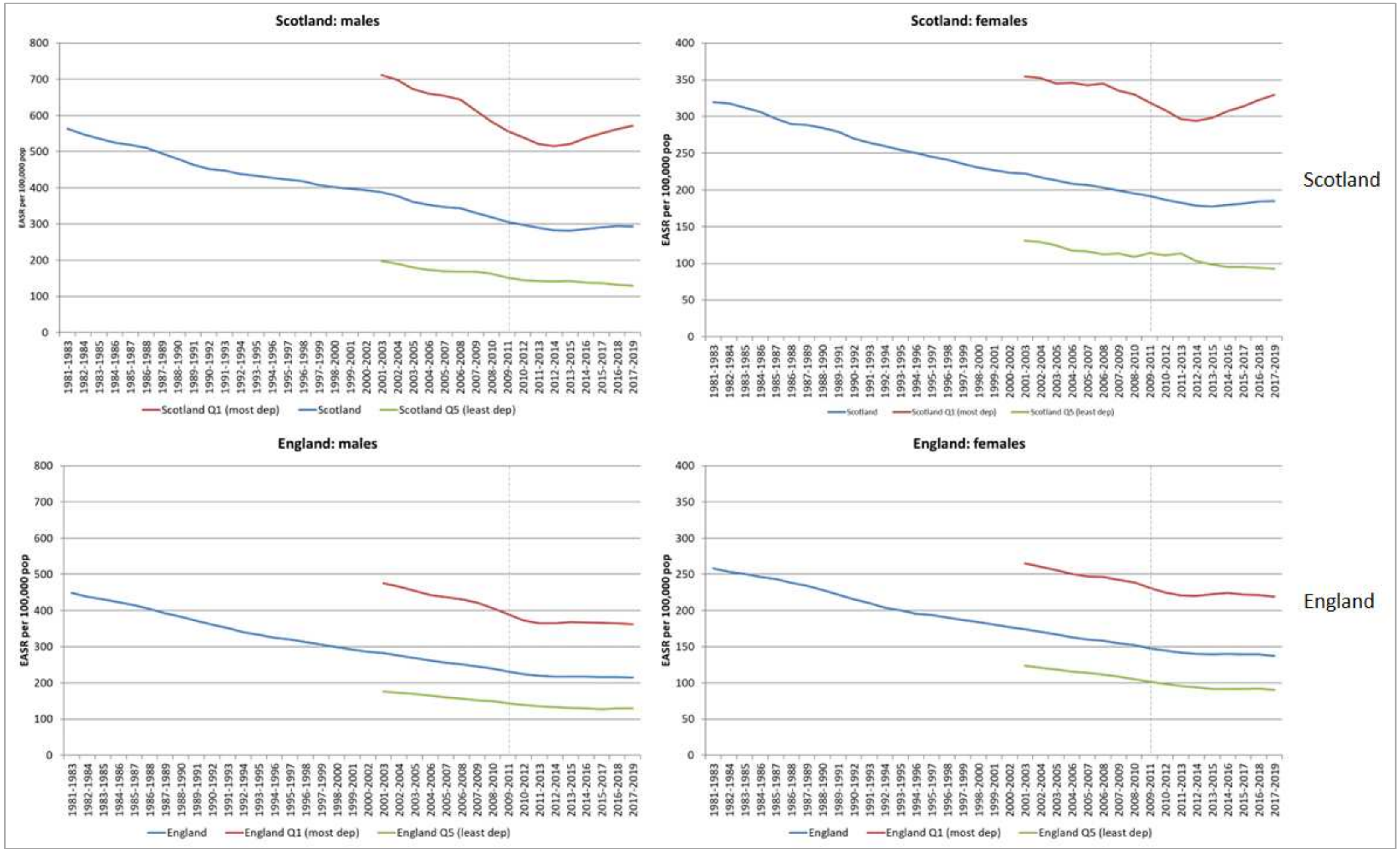

Figure S1. European age-standardised mortality rates per 100,000 population (three-year rolling averages) 1981-2019 for males and females: Scotland, England and their 20% most and least deprived populations (0-64 years). *Note different y-axis scale for males and females*.

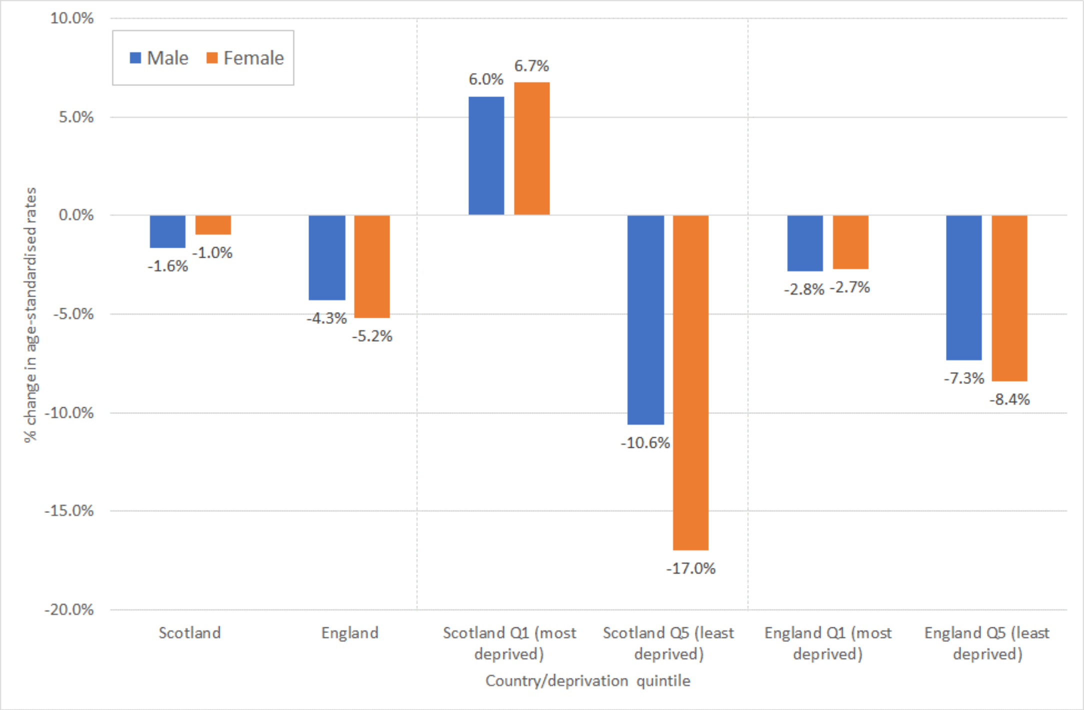

Figure S2. Percentage change in European age-standardised mortality rates (0-64 years), 2010/12 to 2017/19: Scotland, England and their 20% most and least deprived populations

Walsh D, *et al. J Epidemiol Community Health* 2022; 76:1027–1033. doi: 10.1136/jech-2022-219645

a) Scotland

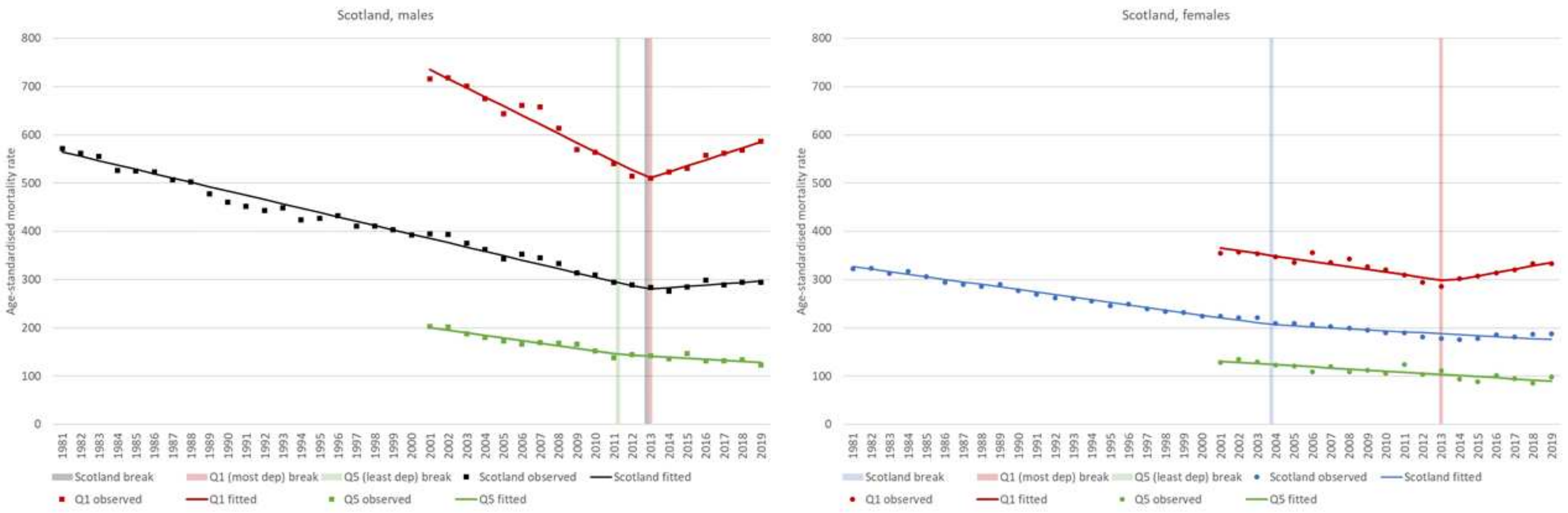
Scotland, males
Age-standardised mortality rate
Scotland break
Q1 (most dep) break
Q5 (least dep) break
Scotland observed
Scotland fitted
Q1 observed
Q1 fitted
Q5 observed
Q5 fitted
Scotland, females
Age-standardised mortality rate
Scotland break
Q1 (most dep) break
Q5 (least dep) break
Scotland observed
Scotland fitted
Q1 observed
Q1 fitted
Q5 observed
Q5 fitted

b) England

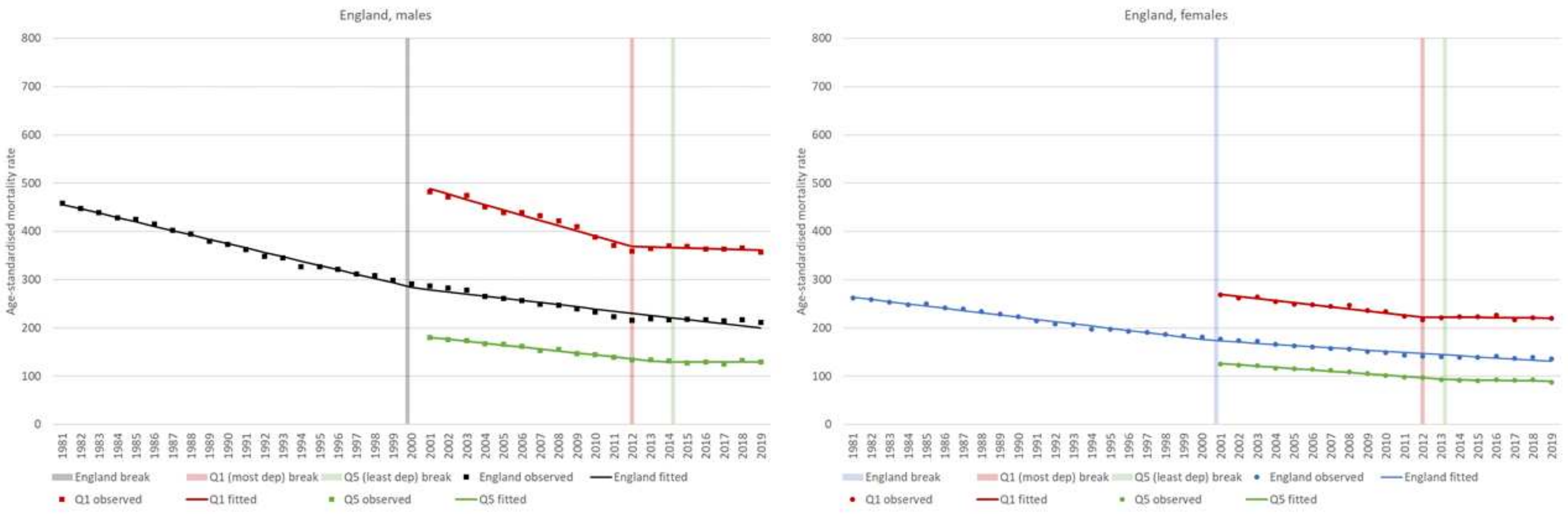

Figure S3. European age-standardised mortality rates (0-64 years), 1981-2019, (a) Scotland (b) England and its 20% most and least deprived populations: observed rates, fitted regression line and break points

Walsh D, *et al. J Epidemiol Community Health* 2022; 76:1027–1033. doi: 10.1136/jech-2022-219645

Table S2. Observed, expected and excess deaths by county and sex, 2012-219 (0-64 years).

Expected deaths based on linear trend 1981-2011; excess deaths shown as percentage of observed deaths.

		Male					Female			
		'Observed'	Expected	Excess (n)	Excess (%)		'Observed'	Expected	Excess (n)	Excess (%)
Scotland	2012	6,294	6,332	**-38**	**-0.6%**		4,006	3,904	**102**	**2.5%**
	2013	6,165	6,137	**27**	**0.4%**		3,921	3,795	**126**	**3.2%**
	2014	5,984	5,947	**37**	**0.6%**		3,894	3,693	**201**	**5.2%**
	2015	6,197	5,775	**422**	**6.8%**		3,931	3,600	**331**	**8.4%**
	2016	6,520	5,610	**910**	**14.0%**		4,116	3,509	**608**	**14.8%**
	2017	6,319	5,436	**884**	**14.0%**		4,020	3,408	**612**	**15.2%**
	2018	6,455	5,251	**1,204**	**18.7%**		4,150	3,304	**846**	**20.4%**
	2019	6,462	5,074	**1,387**	**21.5%**		4,169	3,204	**964**	**23.1%**
	TOTAL 2012-2019	50,396	45,561	**4,834**	**9.6%**		32,206	28,416	**3,790**	**11.8%**
England & Wales	2012	51,171	48,627	**2,545**	**5.0%**		33,401	31,785	**1,616**	**4.8%**
	2013	52,027	46,916	**5,110**	**9.8%**		33,040	30,916	**2,123**	**6.4%**
	2014	51,508	45,295	**6,213**	**12.1%**		33,009	30,101	**2,908**	**8.8%**
	2015	52,308	43,735	**8,574**	**16.4%**		33,286	29,323	**3,963**	**11.9%**
	2016	52,565	42,192	**10,373**	**19.7%**		33,993	28,530	**5,463**	**16.1%**
	2017	52,248	40,485	**11,763**	**22.5%**		33,014	27,697	**5,317**	**16.1%**
	2018	53,124	38,772	**14,352**	**27.0%**		33,702	26,855	**6,848**	**20.3%**
	2019	52,002	36,988	**15,014**	**28.9%**		33,088	25,963	**7,125**	**21.5%**
	TOTAL 2012-2019	416,952	343,010	**73,942**	**17.7%**		266,533	231,169	**35,364**	**13.3%**

Figure S4. 'Observed' vs. expected (based on linear trend 1981-2011) deaths

a) Males, Scotland

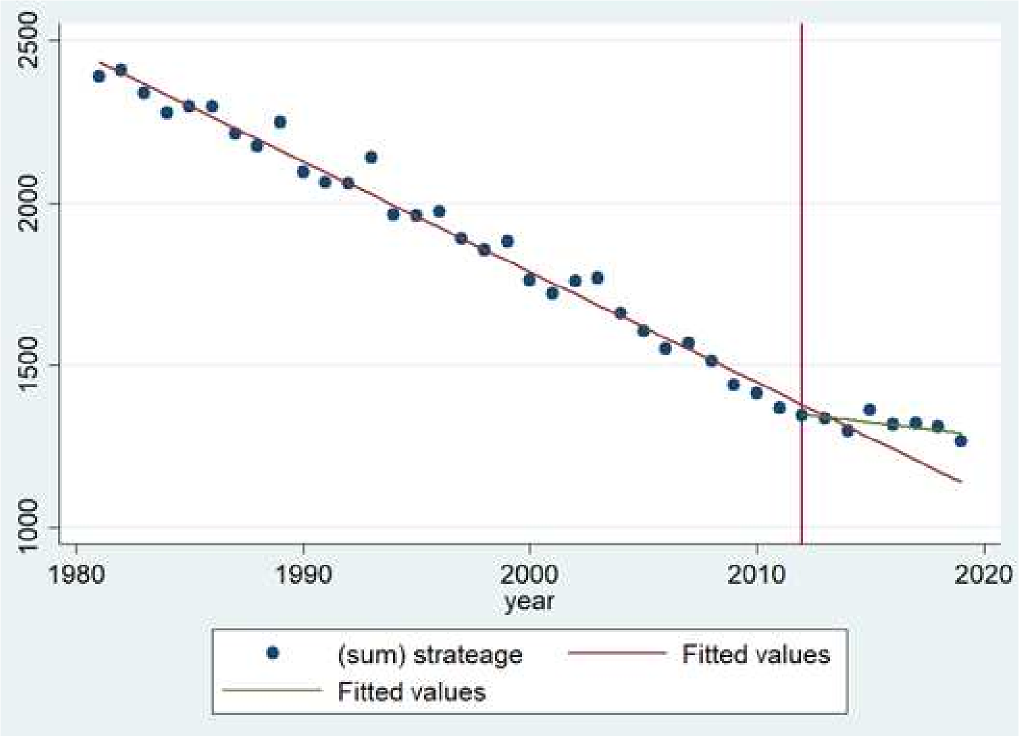

BMJ Publishing Group Limited (BMJ) disclaims all liability and responsibility arising from any reliance placed on this supplemental material which has been supplied by the author(s)

b) Females, Scotland

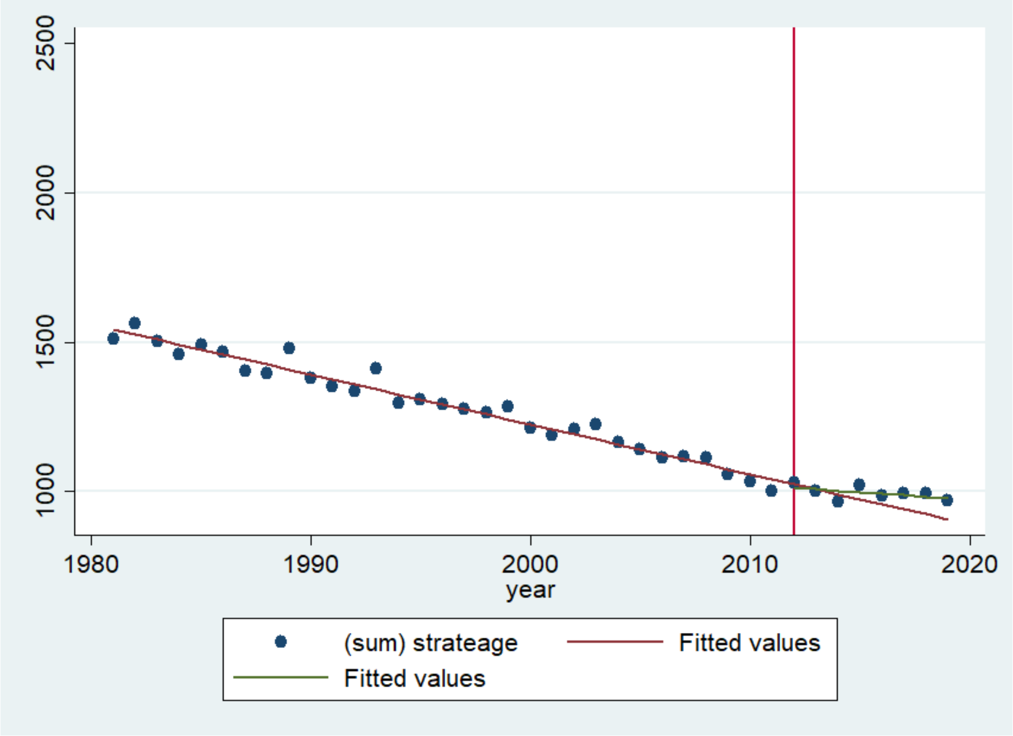

c) Males, England & Wales

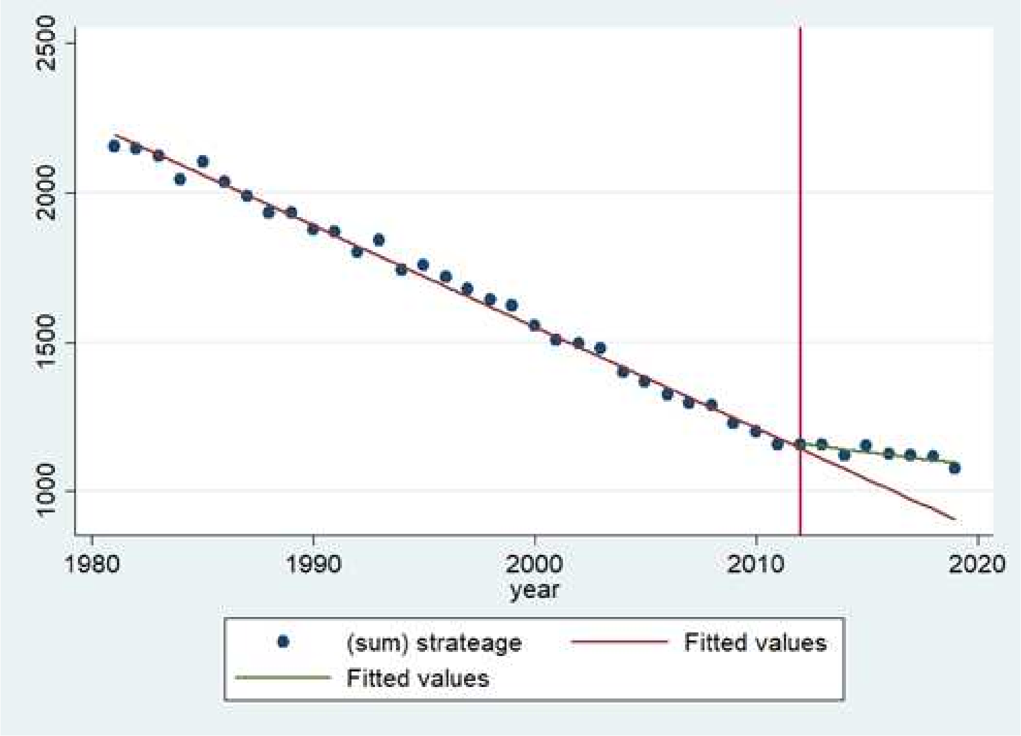

d) Females, England & Wales

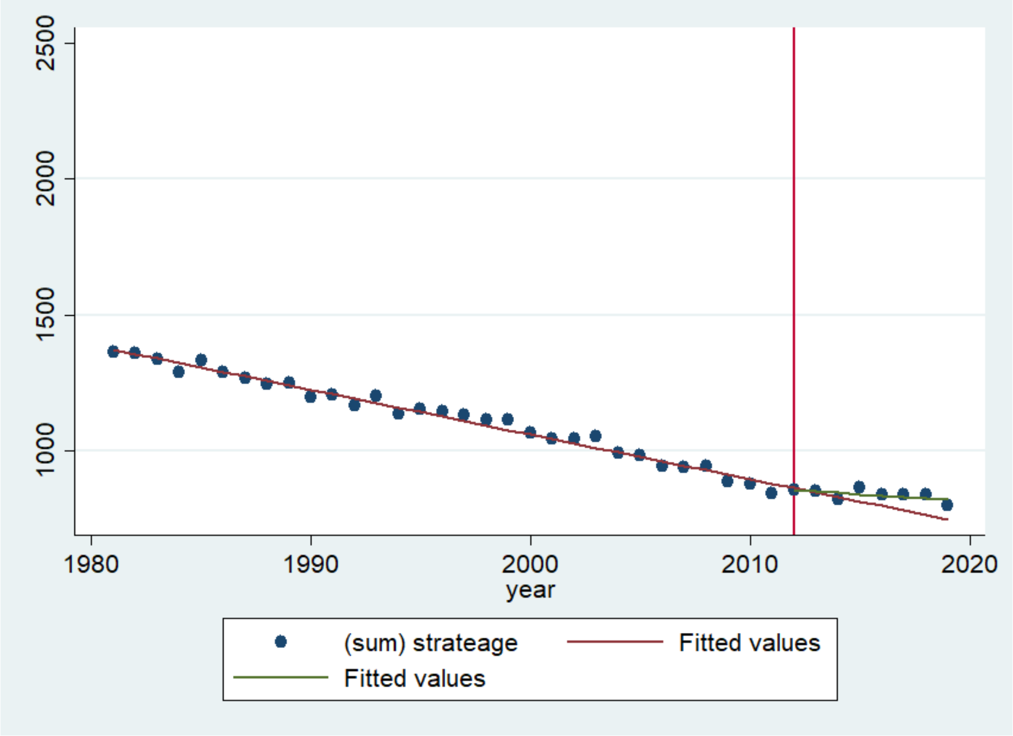

Supplement: Supplementary data [file jech-2022-219645supp001.pdf]
